# *Lactobacillus panisapium* sp. nov., from honeybee *Apis cerana* bee bread

**DOI:** 10.1099/ijsem.0.002538

**Published:** 2018-01-10

**Authors:** Cong Wang, Yan Huang, Li Li, Jun Guo, Zhengyun Wu, Yu Deng, Lirong Dai, Shichun Ma

**Affiliations:** ^1^​ Biogas Institute of Ministry of Agriculture, Chengdu 610041, PR China; ^2^​ College of Light Industry, Textile and Food Engineering, Sichuan University, Chengdu 610065, PR China; ^3^​ Key Laboratory of Development and Application of Rural Renewable Energy, Ministry of Agriculture, Chengdu 610041, PR China; ^4^​ Faculty of Life Science and Technology, Kunming University of Science and Technology, Kunming 650500, PR China

**Keywords:** bee bread, *Lactobacillus*, *Lactobacillus panisapium*, *Apis cerana*

## Abstract

A novel facultatively anaerobic, Gram-stain-positive, non-motile, non-spore-forming, catalase-negative bacterium of the genus *Lactobacillus*, designated strain Bb 2-3^T^, was isolated from bee bread of *Apis cerana* collected from a hive in Kunming, China. The strain was regular rod-shaped. Optimal growth occurred at 37 °C, pH 6.5 with 5.0 g l^−1^ NaCl. The predominant fatty acids were C_18 : 1_ω9*c*, C_16 : 0_ and C_19 : 0_ iso. Respiratory quinones were not detected. Seven glycolipids, three lipids, phosphatidylglycerol and diphosphatidylglycerol were detected. The peptidoglycan type A4α l-Lys-d-Asp was determined. Strain Bb 2-3^T^ was closely related to *Lactobacillus bombicola* DSM 28793^T^, *Lactobacillus apis* LMG 26964^T^ and *Lactobacillus helsingborgensis* DSM 26265^T^, with 97.8, 97.6 and 97.0 % 16S rRNA gene sequence similarity, respectively. A comparison of two housekeeping genes, *rpoA* and *pheS*, revealed that strain Bb 2-3^T^ was well separated from the reference strains of species of the genus *Lactobacillus*. The average nucleotide identity between strain Bb 2-3^T^ and the type strains of closely related species was lower than the 95–96 % threshold value for delineation of genomic prokaryotic species. The G+C content of the genomic DNA of strain Bb 2-3^T^ was 37.4 mol%. On the basis of phenotypic, chemotaxonomic and phylogenetic analyses, strain Bb 2-3^T^ is proposed to represent a novel species of the genus *Lactobacillus*, for which we propose the name *Lactobacillus panisapium* sp. nov. The type strain is Bb 2-3^T^ (=DSM 102188^T^=ACCC 19955^T^).

In recent years, metagenomic analyses and experimental investigations have shown that members of the genus *Lactobacillus* are widely distributed in the gut of honey bees and have positive effects on bee health [1–4]. At the time of writing, there are more than 200 species and subspecies with validly published names (data from EZBioCloud database, https://www.ezbiocloud.net/search?tn=Lactobacillus), but only 11 species (*Lactobacillus apinorum*, *Lactobacillus mellifer*, *Lactobacillus mellis*, *Lactobacillus melliventris*, *Lactobacillus kimbladii*, *Lactobacillus helsingborgensis*, *Lactobacillus bombi*, *Lactobacillus bombicola*, *Lactobacillus apis*, *Lactobacillus kullabergensis* and *Lactobacillus vespulae*) have been isolated from the honey stomach or bee gut [5–9]. Previous studies have revealed that *Lactobacillus* exists in bee bread and plays a key role in its production [10, 11]. In the present study, we characterized a novel species of the genus *Lactobacillus* isolated from the bee bread of *Apis cerana* by using polyphasic and genomics approaches.

During an investigation and comparison of microbial diversity in bee stomach, gut and bread, a novel strain designated Bb 2-3^T^ was isolated from bee bread of *A. cerana* collected from a hive in Kunming, Yunnan province, China. For isolation, the bee bread was mashed and resuspended in sterilized 0.9 % NaCl under anaerobic conditions, followed by serial dilution in anaerobic culture tubes containing sterilized de Man Rogosa and Sharpe (MRS) broth (Thermo Scientific Oxoid) supplemented with 0.5 g l^−1^ l-cysteine, 1.0 mg l^−1^ resazurin and 15 g l^−1^ agar. The MRS medium was prepared and dispensed under a gaseous atmosphere of 100 % nitrogen. Strains were isolated by Hungate roll-tube technique [12, 13]. After incubation for 48 h at 37 °C, a single white, round colony was obtained, designated as strain Bb 2-3^T^, and subjected to taxonomic analysis based on phenotypic and phylogenetic studies.

Extraction and purification of genomic DNA, PCR amplification, and sequencing of the 16S rRNA gene were performed as described previously [14]. All obtained sequences were submitted to NCBI for initial alignment with highly similar sequences using blastn, and 16S rRNA gene sequences of the closely related organisms were retrieved from NCBI (https://www.ncbi.nlm.nih.gov/) and EzTaxon (http://www.ezbiocloud.net/) databases. Housekeeping genes *rpoA* (encoding DNA-directed RNA polymerase subunit alpha) and *pheS* (encoding phenylalanyl-tRNA synthetase subunit alpha) were also used for phylogenetic analysis. *rpoA* and *pheS* gene sequences were retrieved from the genome of strain Bb 2-3^T^. The sequence of *pheS* was confirmed by PCR amplification according to De Bruyne and Snauwaert [7, 15, 16]. Phylogenetic trees were reconstructed with the software package mega version 5.0 using the neighbour-joining and maximum-likelihood methods [17, 18]. The robustness of the topology of the phylogenetic trees was evaluated by bootstrap analysis based on 1000 replications. Phylogenetic analysis based on 16S rRNA gene sequences using the neighbour-joining method revealed that strain Bb 2-3^T^ represented a separated lineage within the genus *Lactobacillus*, together with *L. bombicola* (Fig. 1). Pairwise comparisons of 16S rRNA gene sequences indicated that strain Bb 2-3^T^ was most closely related to *L. bombicola* DSM 28793^T^ (97.8 % 16S rRNA gene sequence similarity), followed by *L. apis* LMG 26964^T^ (97.6 %) and *L. helsingborgensis* DSM 26265^T^ (97.0 %). All other type strains shared less than 97.0 % 16S rRNA gene sequence similarities. Phylogenetic analysis of *rpoA* gene sequences showed that strain Bb 2-3^T^ was closest to *L. apis* LMG 26964^T^ with 91.1 % sequence similarity, followed by *L. helsingborgensis* DSM 26265^T^ (86.6 %) and *L. bombicola* DSM 28793^T^ (84.4 %) (Fig. S1, available in the online version of this article). A *pheS*-based phylogenetic tree reconstructed using the neighbour-joining method indicated that the nearest phylogenetic neighbours to strain Bb 2-3^T^ were *L. apis* LMG 26964^T^ and *L. bombicola*DSM 28793^T^, both sharing 80.1 % sequence similarity (Fig. S2). The phylogenetic position was also confirmed by trees generated using the maximum-likelihood algorithm method (Fig. S3).

**Fig. 1.**
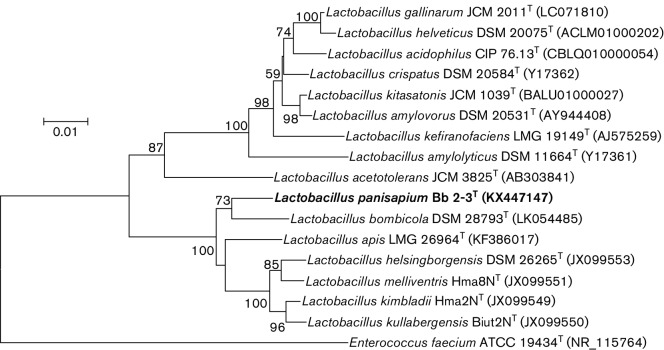
Neighbour-joining phylogenetic tree based on 16S rRNA gene sequences showing the relationship between strain Bb 2-3^T^ and its phylogenetically close relatives. Bootstrap values based on 1000 replications are listed as percentages at the branching points. Bar, 0.01 substitutions per site.

Since the 16S rRNA gene sequence similarity between strain Bb 2-3^T^ and *L. bombicola* DSM 28793^T^, *L. apis* LMG 26964^T^ or *L. helsingborgensis* DSM 26265^T^ exceeded 97 %, genomic relatedness between strain Bb 2-3^T^ and its three phylogenetically closest relatives was determined on the basis of genome sequences. For the comparison of genome relatedness, the genome sequences of strain Bb 2-3^T^, *L. bombicola* DSM 28793^T^ and *L. apis* LMG 26964^T^ were recently sequenced by Majorbio (Shanghai, PR China) using the Illumina MiSeq sequencing platform. All the obtained draft genomes were submitted to the GenBank database. Genome data for *L. helsingborgensis* DSM 26265^T^ was obtained from the GenBank database. The level of pairwise genome-based similarity was evaluated based on both the average nucleotide identity (ANI) value determined by using ChunLab's online ANI calculator (https://www.ezbiocloud.net/tools/ani) described by Yoon *et al*. [19], and the genome-to-genome distance calculation performed by using Genome–Genome Distance Calculator (GGDC) software version 2.1 (http://ggdc.dsmz.de/distcalc2.php) and Formula 2 was used as recommended for the calculation of DDH (DNA–DNA hybridization) for incomplete genomes. The ANI values of strain Bb 2-3^T^ and its related species *L. bombicola* DSM 28793^T^, *L. apis* LMG 26964^T^ and *L. helsingborgensis* DSM 26265^T^ were 76.0, 77.0 and 75.7 %, respectively, which were much lower than the cut-off value of 95–96 %. The digital DDH values between strain Bb 2-3^T^ and *L. bombicola* DSM 28793^T^, *L. apis* LMG 26964^T^ and *L. helsingborgensis* DSM 26265^T^ were 19.9, 20.4 and 18.6 %, respectively, which were lower than the cut-off point of 70 % for the delineation of a novel species. Furthermore, the genomic G+C content was calculated directly from the respective genome sequence and was determined to be 37.4, 34.6, 36.5 and 36.3 mol% for strain Bb 2-3^T^, *L. bombicola* DSM 28793^T^, *L. apis* LMG 26964^T^ and *L. helsingborgensis* DSM 26265^T^, respectively (Table 1). Thus, the phylogenetic and genomic results supported the view that strain Bb 2-3^T^ represents a novel species of the genus *Lactobacillus*.

**Table 1.** Phenotypic comparison of strain Bb 2-3^T^ with its phylogenetically closest relatives Strains: 1, Bb 2-3^T^; 2, *L. bombicola* DSM 28793^T^; 3, *L. apis* LMG 26964^T^; 4, *L. helsingborgensis* DSM 26265^T^. +, Positive; −, negative; L, lipid; GL, glycolipid; PL, phospholipid; PG, phosphatidylglycerol; PGL, phosphoglycolipid; DPG, diphosphatidylglycerol. All data are from this study except where indicated.

**Characteristic**	**1**	**2**	**3**	**4**
Isolation source*	Bee bread of *A. cerana*	Bumble bee gut	Stomach of *A. mellifera*	Stomach of *A. mellifera*
Morphology†	Rods (0.3–0.45×1.5–4.1 µm)	Rods (0.5–1×3.0 µm)	Rods (0.5–1.0×2.1–6.3 µm)	Rods (0.5–0.8×2.0–7.0 µm)
Temperature for growth (°C; optimum)	15–55 (37)	15–55 (37)	15–50 (37)	15–55 (40)
pH for growth (optimum)	3.5–9.0 (6.5)	4.5–8.5 (6.5)	4.0–9.0 (6.5)	4.0–8.5 (6.5)
NaCl tolerance (%, w/v; optimum)	0–8.5 (0.5)	0–4.0 (1.0)	0–6.0 (0.5)	0–5.0 (0.5)
Major cellular fatty acids	C_18 : 1_ω9*c*, C_16 : 0_, C_19 : 0_ iso	C_18 : 1_ω9*c*, C_16 : 0_,	C_18 : 1_ω9*c*, C_16 : 0_,	C_18 : 1_ω9*c*, C_16 : 0_
Polar lipids	L, GL, PG, DPG	L, GL, PL, PG, PGL, DPG	L, GL, PL, PG, DPG	L, GL, PG, PGL, DPG
G+C content (mol%)	37.4	34.6	36.5	36.3
Substrates				
d-Galactose	+	+	−	+
d-Glycogen	−	−	+	+
d-Inositol	−	−	−	+
Lactose	−	−	+	+
d-Mannitol	−	−	+	+
Melibiose	+	−	−	−
d-Ribose	+	+	−	+
d-Sorbitol	−	+	−	+
Sucrose	+	−	+	+
Trehalose	−	+	+	+
d-Xylose	−	−	+	+
(+)-Maltose	−	+	−	−
(+)-Raffinose	+	−	−	+
l-Arabinose	+	+	−	+
l-Rhamnose	+	−	−	+
l-Sorbose	−	−	−	+
Sodium gluconate	−	−	−	+
Main fermentation products (mM)‡				
Lactic acid	87.0	61.7	26.0	10.8
Acetic acid	13.5	0.0	39.6	0.0
Propionic acid	14.1	0.0	0.0	0.0
Butyric acid	8.8	11.1	0.0	0.0

*†Data from [5, 7, 8].

‡Main fermentation products are produced from glucose.

Phenotypic characteristics were determined for strain Bb 2-3^T^ and the reference type strains *L. bombicola* DSM 28793^T^, *L. apis* LMG 26964^T^ and *L. helsingborgensis* DSM 26265^T^. Cell morphology of a 24-hour-old Bb 2-3^T^ culture incubated in MRS medium at 37 °C was examined using a scanning electron microscope (JSM-7500F; JEOL) and transmission electron microscope (JEM-1230; JEOL). Motility was tested using the hanging-drop technique [20]. Gram staining was performed according to Claus *et al*. [21]. Spore-forming ability was examined by phase-contrast microscope (Nikon 80i). Catalase activity, oxidase activity and fermentation type were determined as previously described [7]. Ammonia production from arginine and the Voges–Proskauer test were carried out according to Lopes *et al*. [22]. Hydrolysis of aesculin was tested according to the method described in Tjandraatmadja *et al*. [23]. Hydrolysis of starch was determined based on the formation of clear zones around colonies after applying the suitable staining solutions described previously [24]. H_2_S production was investigated using triple sugar iron agar (BD Difco) supplemented with 2.0 % NaCl. The production of d- and l-lactic acid was analysed using a d-lactic acid/l lactic acid enzyme kit (Magazyme) according to the manufacturer’s instructions. The growth behaviour in the presence of oxygen was determined in a sealed serum bottle containing liquid MRS medium prepared under anaerobic conditions. Nitrogen (0.4, 1.0, 2 or 4 ml) was removed from the 20 ml headspace and the same volume of filter-sterilized oxygen (99.9 %) was added before inoculation to provide 2, 5, 10 or 20 % oxygen. All cultures were incubated without shaking and growth rates were measured.

Growth experiments to determine the pH, temperature, and NaCl concentration ranges were performed in triplicate using anaerobic culture tubes with 5 ml anaerobic MRS medium supplemented with 0.5 g l^−1^ l-cysteine and 1.0 mg l^−1^ resazurin, and the growth rates were measured to evaluate the growth characteristics. The pH range was tested between pH 2.5 and pH 10.0 (at intervals of 0.5 pH units). The sterile anaerobic buffers Na_2_HPO_4_–citric acid (for pH 2.5, 3.0, 3.5, 4.0, 4.5, 5.0 and 5.5), MES (pH 6.0 and 6.5), PIPES (pH 7.0 and 7.5), Tris (pH 8.0 and 8.5), and CHES (pH 9.0, 9.5 and 10.0) were used at a final concentration of 0.02 M to maintain the pH. The temperature range for growth was tested at 10–55 °C at intervals of 5 °C, and the NaCl concentration range was 0–90.0 g l^−1^ (w/v) at intervals of 5.0 g l^−1^. The ability to utilize organic substrates as carbon sources was determined in peptone-yeast extract (PY) medium containing (l^−1^): 0.5 g peptone, 1.0 g yeast extract, 0.5 g trypticase, 4.0 ml salt solution II, 0.5 g l-cysteine and 1.0 mg resazurin. The salt solution II contained the following (g l^−1^): 0.2 CaCl_2_.2H_2_O, 0.48 MgSO_4_.7H_2_O, 1.0 K_2_HPO_4_, 1.0 KH_2_PO_4_, 10.0 NaHCO_3_ and 2.0 NaCl. Cellobiose, d-fructose, d-galactose, d-glucose, d-glycogen, d-inositol, lactose, d-mannitol, melibiose, d-ribose, d-sorbitol, sucrose, trehalose, d-xylose, (+)-maltose, (+)-d-mannose, (+)-raffinose, l-arabinose, l-rhamnose, l-sorbose and sodium gluconate were added to tubes of PY medium as filter-sterilized solution, at a final concentration of 20.0 g l^−1^. Growth was determined using the method as described by Ma *et al*. [25]. Liquid fermentation products were analysed by liquid chromatography (Agilent 1200). CO_2_ was analysed by gas chromatography (Agilent 7820A) [25].

The phenotypic characteristics of strain Bb 2-3^T^ are presented in the species description, Table 1 and Figs S4 and S5. A scanning electron micrograph and transmission electron micrograph of strain Bb 2-3^T^ showed cells were regular-shaped rods with a size of 0.3–0.45×1.5–4.1 µm (Fig. S4). The differential characteristics between strain Bb 2-3^T^ and its closest relatives obtained from the above experiments are listed in Table 1. The key difference between strain Bb 2-3^T^ and its three closest phylogenetic neighbours was the carbohydrate fermentation pattern. Strain Bb 2-3^T^ did not utilize d-glycogen, d-inositol, lactose, d-mannitol, d-sorbitol, trehalose, d-xylose, (+)-maltose, d-sorbose or sodium gluconate. However, strain Bb 2-3^T^ could produce up to 87.0 mM lactic acid when grown on glucose, which was much higher than that of *L. bombicola* DSM 28793^T^ (61.7 mM), *L. apis* LMG 26964^T^ (26.0 mM) and *L. helsingborgensis* DSM 26265^T^ (10.8 mM).

The profiles of cellular fatty acids, respiratory quinones, polar lipids and peptidoglycan structure of strain Bb 2-3^T^, *L. bombicola* DSM 28793^T^, *L. apis* LMG 26964^T^ and *L. helsingborgensis* DSM 26265^T^ were determined by the Identification Service of the Deutsche Sammlung von Mikroorganismen und Zellkulturen (Braunschweig, Germany) using methods described previously [24, 26–31]. Cultures used for measurement of chemotaxonomic characteristics were harvested after cultivation on MRS medium at 37 °C for 18 h.

The cellular fatty acid profile of strain Bb 2-3^T^ comprised C_18 : 1_ω9*c* (54.8 %), C_16 : 0_ (16.2 %) and C_19 : 0_ iso (10.5 %) as the major fatty acids (>10.0 %). The fatty acid profile of the strain was clearly qualitatively and quantitatively different from those of closely related species (Table 2). The polar lipid profile of strain Bb 2-3^T^ comprised three lipids, seven glycolipids, phosphatidylglycerol and diphosphatidylglycerol. Lipids, glycolipids, phosphatidylglycerol and diphosphatidylglycerol were widely distributed in strain Bb 2-3^T^ and its three closest relatives, however, the absence of phospholipid and phosphoglycolipid could distinguish strain Bb 2-3^T^ from its closest relatives within the genus *Lactobacillus* (Fig. S6). Strain Bb 2-3^T^ displayed the peptidoglycan type A4α l-Lys–d-Asp (type A11.31 according to http://www.peptidoglycan-types.info/) in the cell wall, which is identical with that of *L. bombicola* DSM 28793^T^, *L. apis* LMG 26964^T^ and *L. helsingborgensis* DSM 26265^T^. The molar ratio of the amino acids in the peptidoglycan hydrolysate of strain Bb 2-3^T^ was as follows: 1.5 Ala : 0.8 Asp : 1.0 Glu : 0.7 Lys (Table S1).

**Table 2.** Comparison of cellular fatty acid contents (%s) of strain Bb 2-3^T^ with its phylogenetically closest relatives Strains: 1, Bb 2-3^T^; 2, *L. bombicola* DSM 28793^T^; 3, *L. apis* LMG 26964^T^; 4, *L. helsingborgensis* DSM 26265^T^. All data are obtained in this study. Only fatty acids that accounted for >1 % in at least one of the strains are listed. –, Not detected. Fatty acids present at >10 % are highlighted in bold.

**Fatty acid**	**1**	**2**	**3**	**4**
Saturated				
C_14 : 0_	1.3	0.8	0.6	0.9
C_16 : 0_	**16.2**	**15.2**	**13.0**	**18.6**
C_18 : 0_	5.1	3.3	5.5	6.0
Unsaturated				
C_18 : 1_ω9*c*	**54.8**	**69.0**	**64.6**	**63.0**
C_18 : 1_*ω*7*c*	4.9	3.9	5.4	–
Branched-chain				
C_19 : 1_ iso I	1.5	–	1.2	0.6
C_19 : 0_ iso	**10.5**	2.1	7.0	–
Summed features*				
3	1.0	0.7	1.0	1.1
7	2.5	4.0	1.0	4.4
8	4.9	3.9	5.4	4.8

*Summed features represent groups of two or three fatty acids that could not be separated by GLC with the MIDI system. Summed feature 3 comprised C_16 : 1_ω7*c*/C_16 : 1_ω6*c*; summed feature 7 comprised C_19 : 0_ cyclo *ω*10*c*/C_19 : 1_ω6*c*; summed feature 8 comprised C_18 : 1_* ω*7*c*/C_18 : 1_*ω*6*c*.

Therefore, based on phylogenetic, physiological and chemotaxonomic analyses, strain Bb 2-3^T^ represents a novel species of the genus *Lactobacillus*, for which the name *Lactobacillus panisapium* sp. nov. is proposed.

## Description of *Lactobacillus panisapium* sp. nov.

*Lactobacillus panisapium* (pa.nis.a′pi.um. L. masc. n. *panis* bread; L. fem. n *apis* bee; N.L. gen. n. *panisapium* of bee bread).

Cells growing in liquid medium (MRS) under anaerobic conditions are Gram-stain-positive, non-motile, non-spore-forming, long rods with rounded ends, occurring singly, 0.3–0.45 µm wide and 1.5–4.1 µm long, heterofermentative and facultatively anaerobic. The best growth is observed under anaerobic conditions, weaker growth occurs in the presence of oxygen. Colonies on MRS agar under anaerobic conditions after 48 h are round, white and semi-transparent, with a diameter of 2–3 mm. Growth occurs at 15–55 °C (optimum 37 °C), pH 3.5–9.0 (optimum 6.5), and NaCl concentration of 0–85.0 g l^−1^ (optimum 5.0 g l^−1^). Doubling time under the optimal conditions is 0.46 h. Utilizes cellobiose, d-fructose, d-galactose, d-glucose, melibiose, d-ribose, sucrose, (+)-d-mannose, (+)-raffinose, l-arabinose and l-rhamnose. Negative results in tests for starch hydrolysis, ammonia production from arginine, catalase, Voges–Proskauer reaction and H_2_S production, but positive for aesculin hydrolysis. The main fermentation products from glucose are d-/l-lactic acid, acetic acid, propionic acid, butyric acid and CO_2_. The major fatty acids are C_18 : 1_ω9*c*, C_16 : 0_ and C_19 : 0_ iso. Respiratory quinones are not detected. The polar lipid profile is composed of lipids, glycolipids, phosphatidylglycerol and diphosphatidylglycerol. The determined peptidoglycan structure is type A4α l-Lys–d-Asp.

The type strain is Bb 2-3^T^ (=DSM 102188^T^=ACCC 19955^T^), isolated from bee bread of *Apis cerana* collected from a hive in Kunming, China. The genomic DNA G+C content of the type strain is 37.4 mol%.

## Supplementary Data

1206 Supplementary File 1
