# Supplementary material for: Lactobacillus panisapium sp. nov., from honeybee Apis cerana bee bread
Source: Int J Syst Evol Microbiol. 2018 Jan 10;68(3):703–8. doi: 10.1099/ijsem.0.002538 (PMC5882092; doi:10.1099/ijsem.0.002538)
Supplement: Supplementary File 1 [file ijsem-68-703-s001.pdf]

Isolation and characterization of *Lactobacillus panisapium* sp. nov. from honeybee *Apis cerana* bee bread

Cong Wang[1,2,3*], Yan Huang[1,3*], Li Li[2], Jun Guo[4], Zhengyun Wu[2], Yu Deng[1,3], Lirong Dai[1,3], Shichun Ma[1,3†]

1. Biogas Institute of Ministry of Agriculture, Chengdu 610041, P. R. China

2. College of Light Industry, Textile and Food Engineering, Sichuan University, Chengdu 610065, P. R. China

3. Key Laboratory of Development and Application of Rural Renewable Energy, Ministry of Agriculture, Chengdu 610041, P. R. China

4. Faculty of Life Science and Technology, Kunming University of Science and Technology, Kunming 650500, P. R. China

*These authors contributed equally to this work.

[†]Correspondence:

Shichun Ma; mashichun@caas.cn

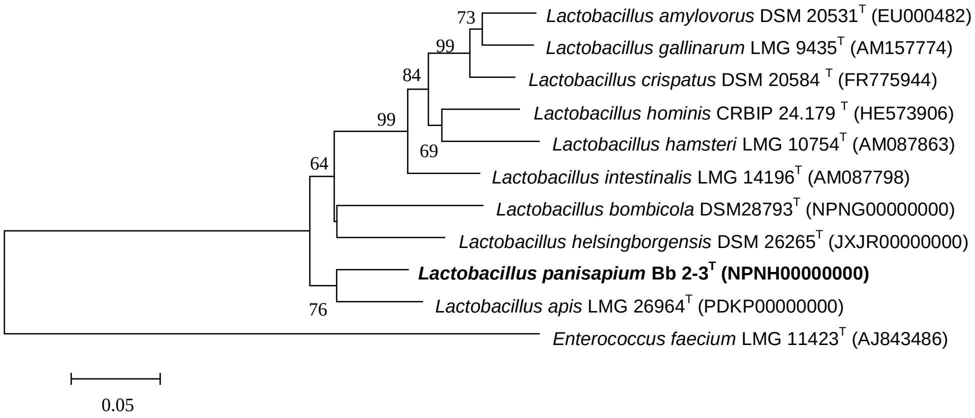

Fig. S1. Neighbour-Joining phylogenetic tree based on *rpoA* gene sequence showing the relationship between strain Bb 2-3^{T} and its phylogenetically close relatives. Bootstrap values based on 1000 replications are listed as percentages at the branching points. Bar, 0.02 substitutions per site. *Enterococcus faecium* LMG 11423^{T} was used as an outgroup.

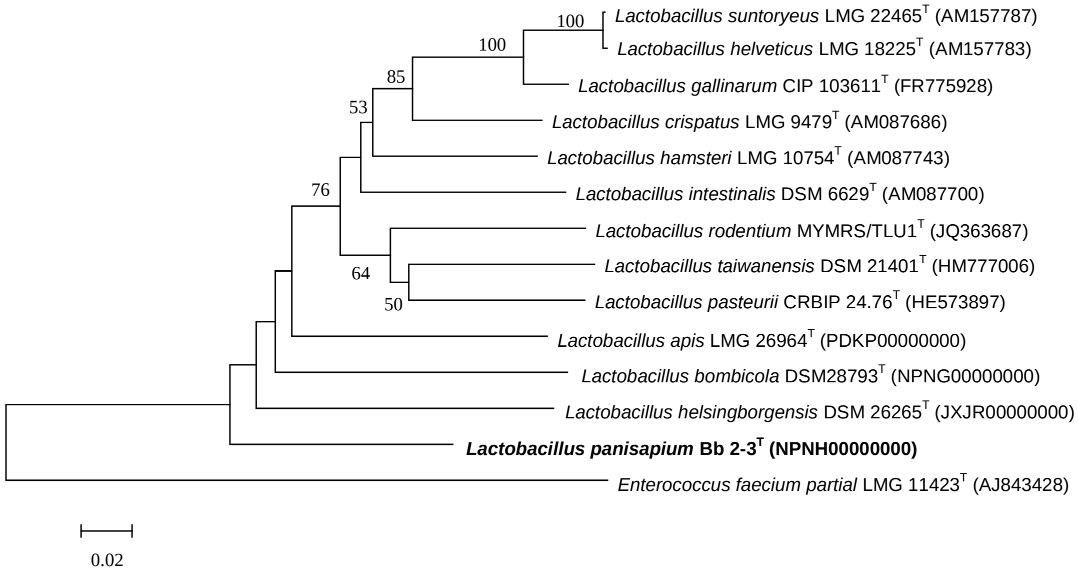

Fig. S2. Neighbour-Joining phylogenetic tree based on *pheS* gene sequence showing the relationship between strain Bb 2-3^{T} and its phylogenetically close relatives. Bootstrap values based on 1000 replications are listed as percentages at the branching points. Bar, 0.02 substitutions per site. *Enterococcus faecium* LMG 11423^{T} was used as an outgroup.

(a)

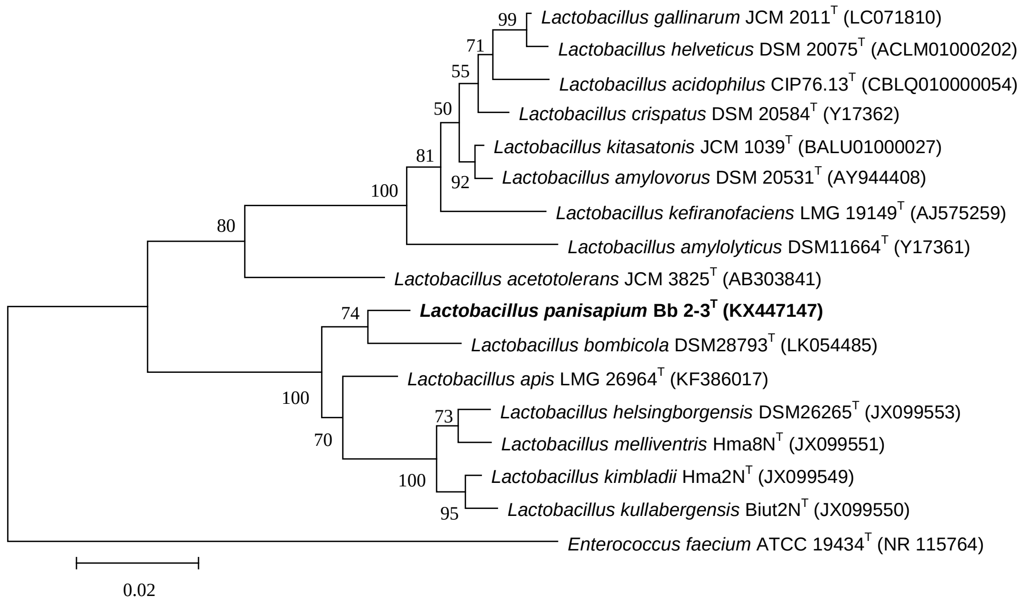

Lactobacillus gallinarum JCM 2011^{T} (LC071810)
Lactobacillus helveticus DSM 20075^{T} (ACLM01000202)
Lactobacillus acidophilus CIP76.13^{T} (CBLQ010000054)
Lactobacillus crispatus DSM 20584^{T} (Y17362)
Lactobacillus kitasatonis JCM 1039^{T} (BALU01000027)
Lactobacillus amylovorus DSM 20531^{T} (AY944408)
Lactobacillus kefiranofaciens LMG 19149^{T} (AJ575259)
Lactobacillus amylolyticus DSM11664^{T} (Y17361)
Lactobacillus acetotolerans JCM 3825^{T} (AB303841)
***Lactobacillus panisapium* Bb 2-3^{T} (KX447147)**
Lactobacillus bombicola DSM28793^{T} (LK054485)
Lactobacillus apis LMG 26964^{T} (KF386017)
Lactobacillus helsingborgensis DSM26265^{T} (JX099553)
Lactobacillus melliventris Hma8N^{T} (JX099551)
Lactobacillus kimbladii Hma2N^{T} (JX099549)
Lactobacillus kullabergensis Biut2N^{T} (JX099550)
Enterococcus faecium ATCC 19434^{T} (NR 115764)
99
71
55
50
81
92
100
80
74
100
73
70
100
95
0.02

(b)

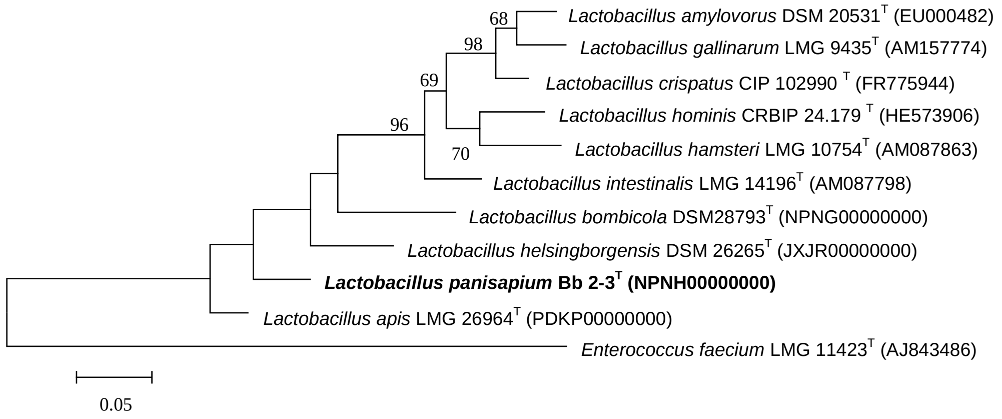

Lactobacillus amylovorus DSM 20531^{T} (EU000482)
Lactobacillus gallinarum LMG 9435^{T} (AM157774)
Lactobacillus crispatus CIP 102990 ^{T} (FR775944)
Lactobacillus hominis CRBIP 24.179 ^{T} (HE573906)
Lactobacillus hamsteri LMG 10754^{T} (AM087863)
Lactobacillus intestinalis LMG 14196^{T} (AM087798)
Lactobacillus bombicola DSM28793^{T} (NPNG00000000)
Lactobacillus helsingborgensis DSM 26265^{T} (JXJR00000000)
***Lactobacillus panisapium* Bb 2-3^{T} (NPNH00000000)**
Lactobacillus apis LMG 26964^{T} (PDKP00000000)
Enterococcus faecium LMG 11423^{T} (AJ843486)
68
98
69
96
70
0.05

(C)

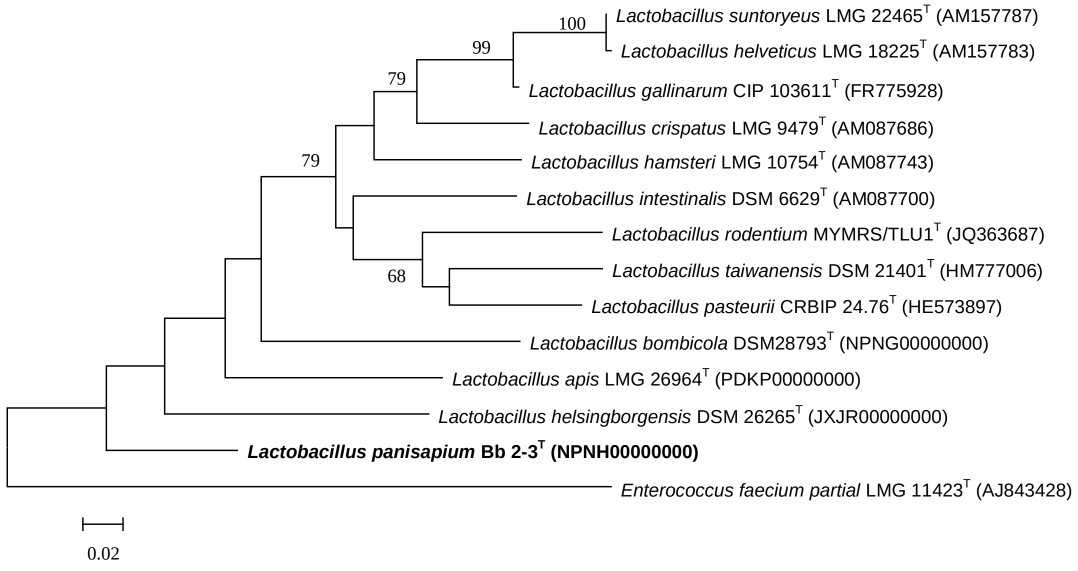

Lactobacillus suntoryeus LMG 22465^{T} (AM157787)
Lactobacillus helveticus LMG 18225^{T} (AM157783)
Lactobacillus gallinarum CIP 103611^{T} (FR775928)
Lactobacillus crispatus LMG 9479^{T} (AM087686)
Lactobacillus hamsteri LMG 10754^{T} (AM087743)
Lactobacillus intestinalis DSM 6629^{T} (AM087700)
Lactobacillus rodentium MYMRS/TLU1^{T} (JQ363687)
Lactobacillus taiwanensis DSM 21401^{T} (HM777006)
Lactobacillus pasteurii CRBIP 24.76^{T} (HE573897)
Lactobacillus bombicola DSM28793^{T} (NPNG00000000)
Lactobacillus apis LMG 26964^{T} (PDKP00000000)
Lactobacillus helsingborgensis DSM 26265^{T} (JXJR00000000)
***Lactobacillus panisapium* Bb 2-3^{T} (NPNH00000000)**
Enterococcus faecium partial LMG 11423^{T} (AJ843428)
100
99
79
79
68
0.02

Fig. S3. Maximum Likelihood phylogenetic tree based on 16S rRNA (a), *rpoA* (b) and *pheS* (c) gene sequences showing the relationship between strain Bb 2-3^{T} and its phylogenetically close relatives. Bootstrap values based on 1000 replications are listed as percentages at the branching points. Bar, 0.02 substitutions per site for a and c, 0.05 substitutions per site for b. Type strain of *Enterococcus faecium* was used as an outgroup.

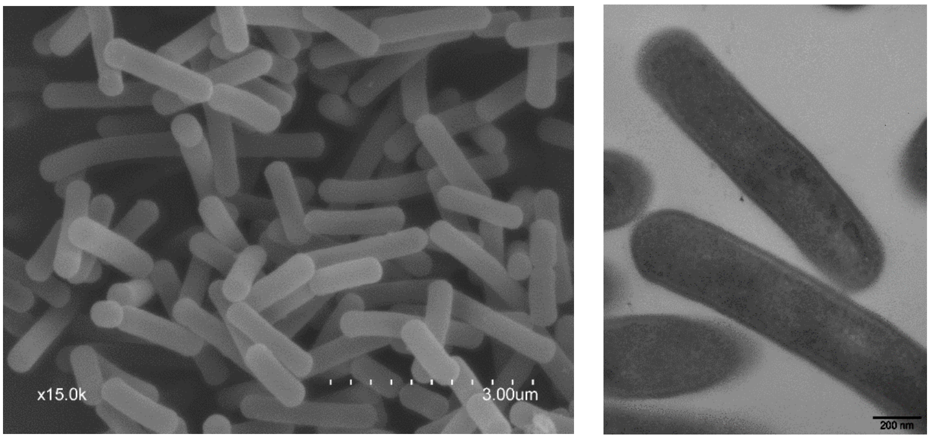

(a) (b)

Fig. S4. Scanning electron micrograph of strain 2-3(a); Transmission electron micrograph of thin sections of cells cultured for 24 h(b).

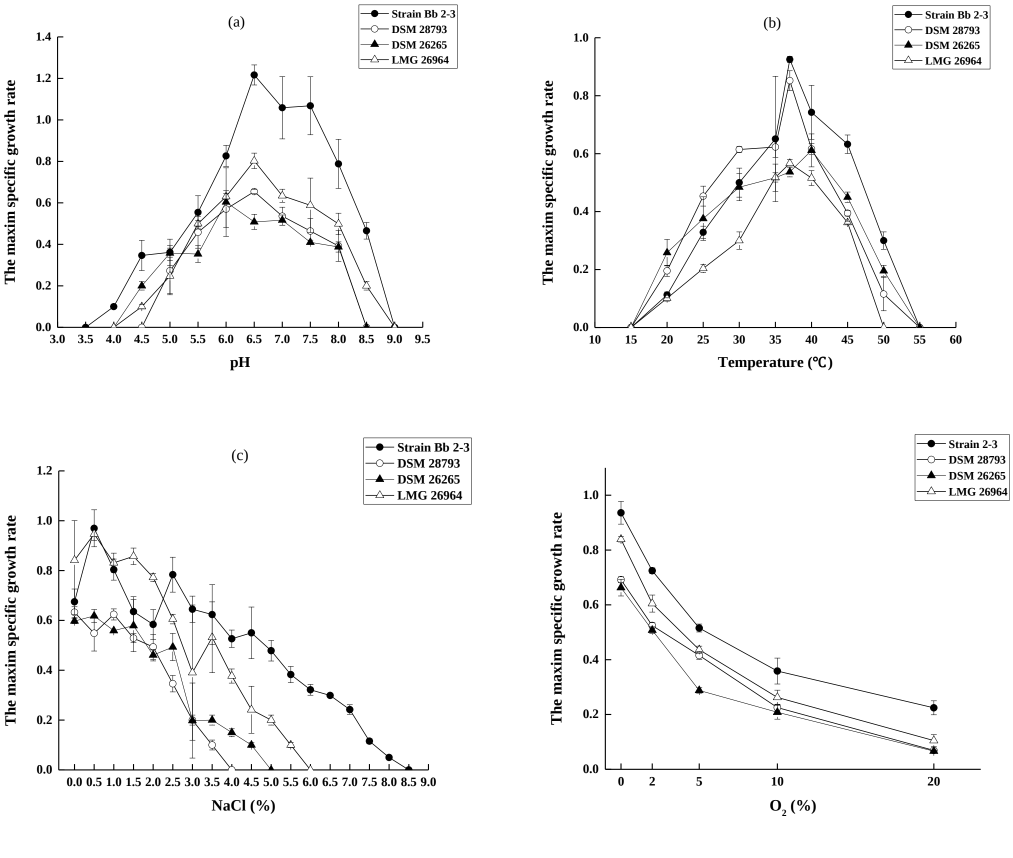

Fig. S5. Effects of pH (a), temperature (b), NaCl concentration (c), and O_2 (%) on the growth of strain Bb 2-3^{T} and closely related strains.

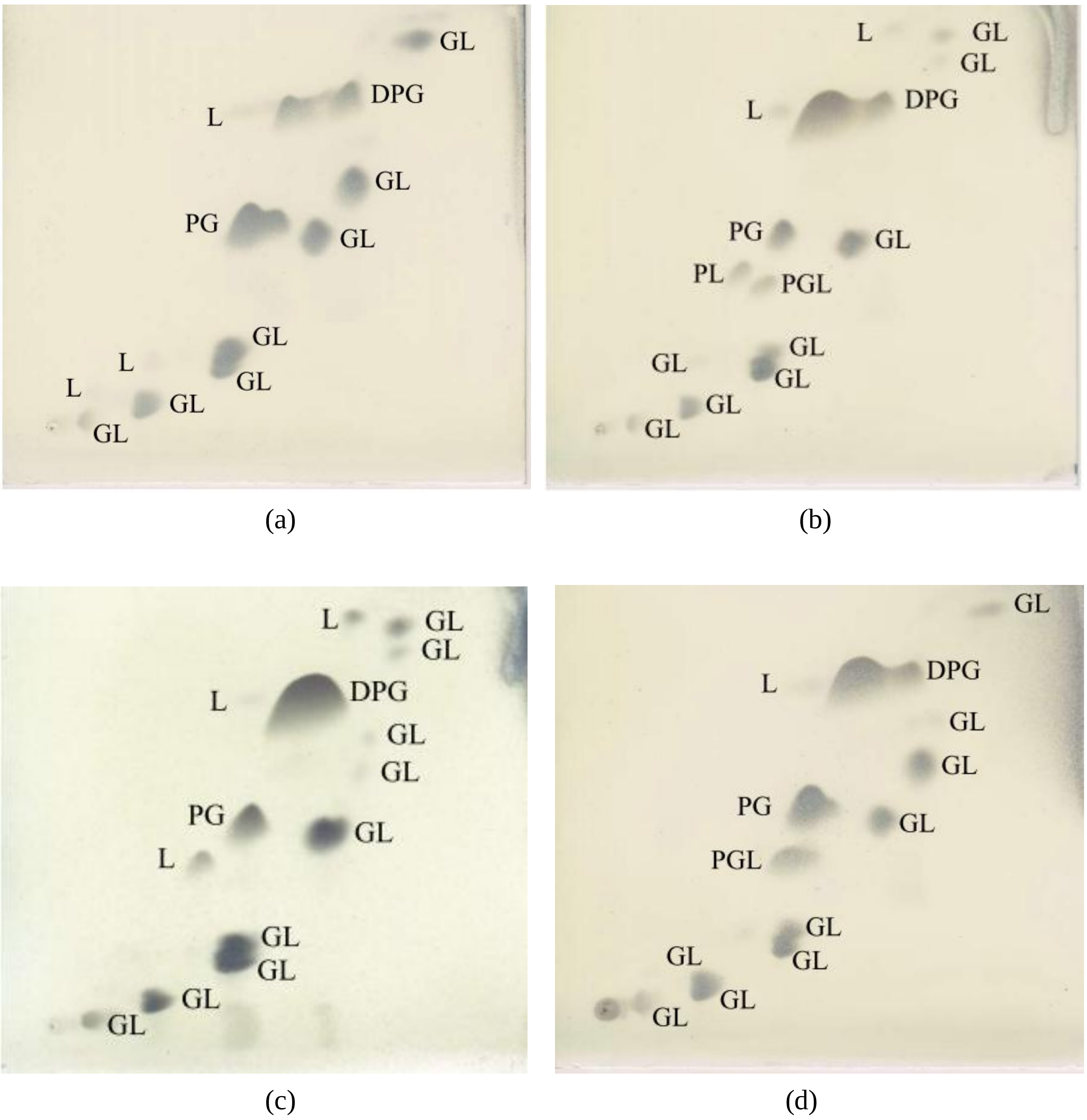

Fig. S6. Polar lipid profile of strain Bb 2-3^T and closely related strains.

(a) strain Bb 2-3^T; (b) *L. bombicola* DSM 28793^T; (c) *L. apis* LMG 26964^T (d) *L. helsingborgensis* DSM 26265^T; L = lipid, GL = glycolipid, PL = phospholipid, PG = phosphatidylglycerol, PGL = Phosphoglycolipid, DPG = diphosphatidylglycerol.

Table S1. Peptidoglycan structure of strain Bb 2-3^T and related taxa of the species within *Lactobacillus*.

Strain	Peptidoglycan structure	Molar ratio of amino acids in the peptidoglycan hydrolysate
Bb 2-3^T	A4α L-Lys–D-Asp type	1.5 Ala:0.8 Asp:1.0 Glu:0.7 Lys
L. bombicola DSM 28793^T	A4α L-Lys–D-Asp type	1.7 Ala:0.8 Asp:1.0 Glu:0.6 Lys
L. apis LMG 26964^T	A4α L-Lys–D-Asp type	1.5 Ala:0.8 Asp:1.0 Glu:0.7 Lys
L. helsingborgensis DSM 26265^T	A4α L-Lys–D-Asp type	1.7 Ala:0.3 Asp:1.0 Glu:0.3 Lys
